# The therapeutic effects of attending a one-day outpatient service on patients with gestational diabetes and different pre-pregnancy body mass indices

**DOI:** 10.3389/fpubh.2022.1051582

**Published:** 2023-03-07

**Authors:** Yan-Min Cao, Min Ma, Wei Wang, Na-Na Cai

**Affiliations:** Department of Internal Medicine, The Fourth Hospital of Shijiazhuang, Key Laboratory of Maternal and Fetal Medicine of Hebei Provincial, Shijiazhuang, China

**Keywords:** gestational diabetes mellitus, one-day clinic, body mass index, gestational weight gain, blood sugar

## Abstract

**Purpose:**

This study investigated the effects of attending a one-day outpatient service on the outcomes of patients with gestational diabetes mellitus (GDM) and different pre-pregnancy body mass indices (BMIs).

**Methods:**

The study recruited 311 pregnant women with GDM into a one-day outpatient service at The Fourth Hospital of Shijiazhuang from September 2019 to December 2021. They were randomly assigned to three groups, based on their pre-pregnancy BMI as follows: group A, BMI < 18.5 kg/m^2^; group B, 18.5 ≥ BMI > 25.0 kg/m^2^; group C, BMI ≥25 kg/m^2^. The following information was collected from all the participants: fasting blood glucose, hemoglobin A1c (HbA1C), insulin dose, gestational weight gain, weight gain after the one-day outpatient service, and perinatal outcomes.

**Results:**

The three groups showed significant differences in fasting blood glucose and HbA1C, insulin treatment rate, and the incidence of pregnancy hypertension/preeclampsia and neonatal jaundice (all *P* < 0.05). The rate of excessive gestational weight gain in all of the groups also reflected significant differences (*P* < 0.05). Group A showed the lowest weight gain, while group C gained the most weight. There is no significant difference in the incidences of hypertension/preeclampsia, neonatal jaundice, or premature birth between patients with weight loss/no weight gain and those with positive weight gain.

**Conclusion:**

One-day diabetes outpatient integrated management may effectively help to manage weight gain and blood glucose in patients with GDM and different pre-pregnancy BMIs. Dietary control after a GDM diagnosis may have helped to avoid weight gain entirely, as well as negative weight gain, but did not increase the risk of maternal and infant-related complications.

## Introduction

Gestational diabetes mellitus (GDM) is the one of the most common complications during pregnancy. It is commonly defined as the very first recognition of diabetes or impaired glucose tolerance during pregnancy. However, there is no universally accepted protocol to diagnose GDM (1–3). According to the report of the International Diabetes Federation (IDF) in 2019, 16.7% of live births were affected by hyperglycemia in pregnant women and 84% of them had gestational diabetes (4). GDM has gradually become a global health concern. The prevalence of GDM was the highest in Middle East and North Africa, followed by Southeast Asia, Western Pacific regions, and even in the developed areas, such as North America (5). Approximately 10% of pregnant women in Asia developed GDM, and the prevalence of GDM was much higher in low- and upper-middle areas (6). GDM is associated with many other complications in pregnant women. Existing studies indicated that GDM was closely related to type 2 diabetes (7, 8). Moreover, in 2011, the American Heart Association classified GDM as a risk factor for the development of cardiovascular disease in women (9). GDM not only impairs the health of pregnant women but also potentially raises the risk of disease in the offspring. Several studies have shown that women who had been diagnosed with GDM were more likely to give birth to offspring with hypertension, meanwhile, high blood glucose levels during pregnancy were shown to alter the metabolism of neonates, leading to adiposity (10, 11). Therefore, the effective management and treatment of patients with GDM are of great significance to maternal and infant health and prognosis.

A meta-analysis showed a pooled estimated risk of GDM in pregnant women of 16.8%, with a risk of 10.7% in the underweight/normal group, and 23% in the overweight/obesity group, suggesting an elevated risk of GDM in pregnant women who are overweight/obese (12). One-day outpatient management of diabetes enables patients with GDM to learn effective self-management strategies by providing them with information about diet control and exercise therapy, as well as one-to-one supervision and guidance using the WeChat application. By actively following a doctor's advice regarding diet control and exercise, patients can effectively manage their blood glucose levels and reduce adverse pregnancy outcomes. This study aimed to identify the differences in blood glucose control, perinatal outcomes, and weight change during pregnancy among participants with GDM who had different pre-pregnancy body mass indices (BMIs). Additionally, the study assessed the impact of attending a comprehensive one-day diabetes outpatient management program on gestational weight change and pregnancy-related outcomes.

## Materials and methods

### Study design

This observational study included 311 pregnant women with GDM who participated in a one-day diabetes outpatient program at the authors' hospital from September 2019 to December 2021. All participants were of Chinese Han ethnicity. All participants provided a signed informed consent form for their inclusion in the study.

Body mass index is an internationally used body-fat and health measurement tool and is calculated using the following formula: BMI = weight/height^2^, with weight in kg and height in meters. The participants were divided into three groups, based on their BMI prior to becoming pregnant as follows: group A represented the low weight group (BMI < 18.5 kg/m^2^ before pregnancy), group B reflected the normal weight group (18.5 ≤ BMI < 25.0 kg/m^2^ before pregnancy), and group C represented the overweight and obese group (BMI ≥25 kg/m^2^ before pregnancy). Age, height, family history of diabetes, gestational age, and other patient characteristics were not significantly different (*P* > 0.05), indicating comparability among the three BMI groups. After attending the one-day outpatient service, the pregnant women with GDM were classified according to positive, negative, or no weight gain.

### Inclusion and exclusion criteria

The following criteria were based on guidelines issued by the American Diabetes Association (13). All participants included in this study had been newly diagnosed with GDM using a 75 g oral glucose tolerance test (OGTT). The study's exclusion criteria were as follows: (1) fasting plasma glucose ≥7.0 mmol/L; (2) OGTT 2-h blood glucose ≥11.1 mmol/L; (3) typical symptoms of hyperglycemia or hyperglycemia crisis accompanied by an arbitrary blood glucose level ≥11.1 mmol/L; (4) hemoglobin A1c (HbA1c) ≥6.5%, indicative of pre-pregnancy diabetes combined with pregnancy; (5) gemellary or multiple pregnancy.

### One-day outpatient service

The one-day outpatient service is an umbrella program that includes the following services: (1) introducing a healthy diet and how to cook scientifically; (2) guiding patients on how to appropriately and individually design an exercise plan; (3) conducting introductory courses in the management of weight and blood glucose, and in addition, completing a quiz; (4) creating a friendly environment for instructors and patients in which to communicate with each other; (5) setting up a WeChat group to monitor and follow up the patients.

The authors conducted the one-day outpatient training twice per week, each time with a small class of ~10 participants. An endocrinologist and a diabetes nurse were responsible for delivering this training. In the morning, participants underwent physical examinations and were given diabetes-friendly meals. The endocrinologist educated the participants about exercise and delivered introductory courses. Through these courses, the participants were able to learn about a GDM diagnosis and recognize its related risk factors. The courses enabled the current authors to help participants design more scientific and healthier diets and exercise plans, and they were also trained on how to monitor their blood glucose levels and other related simple symptoms on their own at home. The participants were reminded to conduct retesting once they had given birth. Counseling services were available to participants if necessary. In the afternoon, they watched childbirth and breastfeeding videos, and the study authors gave them one-on-one instructions. After finishing all the training sessions, the authors presented a questions-and-answers session. Furthermore, a quiz was conducted to test how much the participants had learned from the one-day outpatient service, and a questionnaire subsequently assisted in adjusting and improving the one-day outpatient service. During the entire day of training, participants completed multiple blood glucose tests before and after diets. Notably, WeChat groups were created at the end of the one-day program *via* which the authors regularly posted GDM-related information and reminded participants to regularly retest themselves. The authors also answered participants' questions *via* the application to help them gain a better understanding of their health condition.

### Statistical analysis

The SPSS Statistics 26.0 software program was used to analyze the collected data. Continuous variables were described using mean ± standard deviation and ANOVA was used to compare the mean values among three groups. Categorical data were expressed as a percentage (%) and compared using chi-square (χ^2^) test. Given that the sample size was >30, and based on existing studies (14, 15), the statistical significance was set as *P* < 0.05, and all tests were two-sided.

## Results

### Demographic characteristics

A total of 320 pregnant women were screened and 9 of them were excluded due to gemellary pregnancy. Finally, 311 patients were enrolled. The age, height, family history of diabetes, and gestational age were not significantly different among the three groups (*P* > 0.05) (Table 1).

**Table 1:** Comparison of the general data among the three groups (X ± s).

**Items**	**A (*n* = 18)**	**B (*n* = 183)**	**C (*n* = 110)**	***P***
Age (years)	30.33 ± 3.73	30.43 ± 4.07	31.01 ± 4.38	0.49
Height (cm)	161.3 3± 4.55	161.36 ± 4.71	161.66 ± 4.85	0.86
Pregnancy week at entry (weeks)	26.00 ± 1.19	26.13 ± 1.34	26.03 ± 1.30	0.07
Family history of diabetes — no./total no. (%)	8/18 (44.4)	61/183 (33.3)	36/110 (32.7)	0.65

A: low weight group (body mass index [BMI] < 18.5 kg/m^2^ before pregnancy), B: normal weight group (18.5 ≤ BMI < 25.0 kg/m^2^ before pregnancy), C: overweight and obesity group (BMI ≥ 25 kg/m^2^ before pregnancy).

### Comparison of blood glucose changes among the three groups after the intervention

Fasting blood glucose and HbA1C were significantly different among the three groups at enrollment and before delivery (*P* < 0.05), where group A had the lowest and group C had the highest fasting blood glucose and HbA1C levels. No significant differences were observed in fasting blood glucose and HbA1C among the three groups before delivery (*P* > 0.05). Meanwhile, the insulin treatment rate was significantly different among the three groups (*P* < 0.05), where group A had the lowest and group C had the highest insulin treatment rate (Table 2).

**Table 2:** Comparison of blood glucose changes among the three groups ($\bar{X}$ ± S).

**Items**		**A (*n* = 18)**	**B (*n* = 183)**	**C (*n* = 110)**	***P***
Fasting glucose (mmol/L)	At entry	5.08 ± 0.38	5.25 ± 0.46	5.52 ± 0.43	< 0.001
	Before delivery	4.66 ± 0.35	4.77 ± 0.41	4.93 ± 0.47	< 0.001
	At entry to before delivery	0.43 ± 0.33	0.49 ± 0.50	0.60 ± 0.52	0.11
HbA1C (%)	At entry	5.09 ± 0.37	5.18 ± 0.33	5.24 ± 0.37	< 0.001
	Before delivery	5.18 ± 0.40	5.31 ± 0.34	5.49 ± 0.44	< 0.001
	At entry to before delivery	−0.09 ± 0.21	−0.14 ± 0.26	−0.13 ± 0.27	0.78
Insulin-treated women, no./total no. (%)		0/18 (0.0)	10/183 (5.5)	15/110 (13.6)	0.02

A: low weight group (body mass index [BMI] < 18.5 kg/m^2^ before pregnancy), B: normal weight group (18.5 ≤ BMI < 25.0 kg/m^2^ before pregnancy), C: overweight and obesity group (BMI ≥ 25 kg/m^2^ before pregnancy).

### Comparison of pregnancy outcomes among the three groups after the intervention

The incidences of pregnancy, hypertension/preeclampsia, and neonatal jaundice were significantly different among the three groups (*P* < 0.05), where group A had the lowest and group C had the highest incidences. No significant differences were found in the incidence of premature membrane rupture, premature delivery, polyhydramnios/oligohydramnios, neonatal hypoglycemia, fetal distress, macrosomia, fetal growth restriction, or cesarean section rate among the three groups (*P* > 0.05) (Table 3).

**Table 3:** Comparison of pregnancy outcomes among the three groups (X ± S).

**Items**	**A (*n* = 18)**	**B (*n* = 183)**	**C (*n* = 110)**	***P***
Gestational hypertension/preeclampsia, no./total no. (%)	0/18 (0.0)	9/183 (4.9)	22/110 (20.0)	< 0.001
Women with PMR, no./total no. (%)	5/18 (27.8)	34/183 (18.6)	20/110 (18.2)	0.62
Preterm birth, no. /total no. (%)	3/18 (16.7)	11/183 (6.0)	15/110 (13.6)	0.05
Women with hydramnios/oligohydramnios, no./total no. (%)	0/18 (0.0)	11/183 (6.0)	3/110 (2.7)	0.40
Newborn babies with jaundice, no./total no. (%)	0/18 (0.0)	42/183 (23.0)	38/110 (34.5)	< 0.001
Newborn babies with hypoglycemia, no. /total no. (%)	1/18 (5.6)	6/183 (3.3)	1/110 (0.9)	0.19
Fetal distress, no. /total no. (%)	1/18 (5.6)	21/183 (11.5)	9/110 (8.2)	0.57
Macrosomia, no. /total no. (%)	0/18 (0.0)	9/183 (4.9)	9/110 (8.2)	0.26
Fetal growth restriction, no./total no. (%)	0/18 (0.0)	2/183 (1.1)	1/110 (0.9)	1.00
Cesarean delivery rate, no./total no. (%)	5/18 (27.8)	77/183 (42.1)	58/110 (52.7)	0.07

A: low weight group (body mass index [BMI] < 18.5 kg/m^2^ before pregnancy), B: normal weight group (18.5 ≤ BMI < 25.0 kg/m^2^ before pregnancy), C: overweight and obesity group (BMI ≥25 kg/m^2^ before pregnancy).

PMR, premature membrane rupture.

### Comparison of body weight changes among the three groups after the intervention

The overall weight change and excess weight-gain rate during pregnancy were significantly different among the three groups (*P* < 0.05), where group A had the lowest and group C had the highest overall weight change and excess weight-gain rate during pregnancy. No statistically significant differences were observed in negative weight-gain rate, underweight-gain rate, or normal weight-gain rate (*P* > 0.05). Body- weight changes were significantly different among the three groups after admission to a one-day outpatient clinic (*P* < 0.05), where group A had the lowest negative weight gain and a zero weight-gain rate, while group C had the highest, including three participants with negative weight gain. The positive weight-gain rate was the highest in group A and the lowest in group C (Table 4).

**Table 4:** Comparison of body weight changes among the three groups (X ± S).

**Items**		**A (*n* = 18)**	**B (*n* = 183)**	**C (*n* = 110)**	***P***
Changes in total weight during pregnancy	Negative rate of weight gain (%)	0/18 (0.0)	0/183 (0.0)	3/110 (2.7)	0.07
	Underweight gain rate (%)	7/18 (38.9)	84/183 (45.9)	40/110 (36.4)	0.29
	Normal rate of weight gain (%)	9/18 (50.0)	70/183 (38.3)	34/110 (30.9)	0.23
	Excessive weight gain rate (%)	2/18 (11.1)	29/183 (15.8)	33/110 (30.0)	0.01
Body weight changes after admission to the one-day clinic	Negative weight gain and no weight gain rate (%)	1/18 (5.6)	30/183 (16.4)	32/110 (29.1)	0.01
	Positive rate of weight gain (%)	17/18 (94.4)	153/183 (83.6)	78/110 (70.9)	

A: low weight group (body mass index [BMI] < 18.5 kg/m^2^ before pregnancy), B: normal weight group (18.5 ≤ BMI < 25.0 kg/m^2^ before pregnancy), C: overweight and obesity group (BMI ≥25 kg/m^2^ before pregnancy).

### The influence of weight gain on pregnancy outcomes after the one-day outpatient clinic

The incidences of pregnancy, hypertension/preeclampsia, premature membrane rupture, premature childbirth, polyhydramnios/oligohydramnios, neonatal jaundice, neonatal hypoglycemia, fetal distress, macrosomia, fetal growth restriction, and cesarean section rate were not significantly different among the negative weight gain, no weight gain, and positive weight gain groups (*P* > 0.05) (Table 5).

**Table 5:** Influence of weight change on pregnancy outcomes after 1 day of the outpatient clinic (X ± S).

**Items**	**Negative weight gain and no weight gain (*n* = 63)**	**Positive growth (*n* = 248)**	***P***
Gestational hypertension/preeclampsia, no./total no. (%)	4/63 (6.3)	27/248 (10.9)	0.28
Women with PMR, no./total no. (%)	7/63 (11.1)	52/248 (21.0)	0.08
Preterm birth, no./total no. (%)	8/63 (12.7)	21/248 (8.5)	0.33
Women with hydramnios/oligohydramnios, no./total no. (%)	3/63 (4.8)	11/248 (4.4)	1.00
Newborn babies with jaundice, no./total no. (%)	20/63 (31.7)	60/248 (24.2)	0.22
Newborn babies with hypoglycemia, no./total no. (%)	0/63 (0.0)	8/248 (3.2)	0.32
Fetal distress, no./total no. (%)	5/63 (7.9)	26/248 (10.5)	0.55
Macrosomia, no./total no. (%)	3/63 (4.8)	15/248 (6.0)	0.93
Fetal growth restriction, no./total no. (%)	1/63 (1.6)	2/248 (0.8)	0.49
Cesarean delivery rate, no./total no. (%)	27/63 (42.9)	113/248 (45.6)	0.70

PMR, premature membrane rupture.

## Discussion

Weight gain and obesity are critical risk factors for the development of diabetes. A cross-sectional study including 31 provinces and cities in Mainland China showed that the prevalence rates of diabetes and prediabetes in women of childbearing age in China between 2010 and 2012 were 1.4 and 12.9%, respectively. Meanwhile, incidences of being overweight (BMI 25.0–29.9 kg/m^2^) and obese (BMI ≥30 kg/m^2^) were 7.2 and 1.0%, respectively (16). Being overweight or obese before becoming pregnant poses an independent risk for GDM development. A multivariate logistic regression study discovered that pre-pregnancy BMI was significantly associated with maternal hyperglycemia (17). Similarly, another meta-analysis revealed that the incidence of GDM was significantly higher in obese women before pregnancy than in the normal BMI group, regardless of weight gain, and that the incidence of GDM increased by 0.92% (95% confidence interval, 0.73–1.10) for every 1-unit increase in BMI (18). Excessive weight gain during pregnancy increased the risk of infants presenting as large for gestational age (LGA), macrocephaly, cesarean section, hypertension during pregnancy, postpartum hemorrhage, and other adverse events. Even among women with a normal BMI before pregnancy, excessive gestational weight gain increased the risk of postpartum hemorrhage and LGA babies (19). Therefore, pre-pregnancy weight and weight gain during pregnancy are critical for patients with GDM.

The one-day outpatient service was implemented to manage patients' weight gain using a science-based diet and proper exercise, to empower patients to effectively control their blood glucose levels, and to reduce maternal and infant-related complications. This study observed the therapeutic effects of attending the one-day outpatient service on participants with GDM who had different pre-pregnancy BMIs. The study also assessed the impacts of different weight-gain levels during gestation on pregnancy-related outcomes.

The study findings demonstrated that fasting blood glucose was lowest in the low weight group at enrollment and before delivery but was slightly higher in the normal weight group and highest in the overweight and obesity group. This difference was statistically significant among the three groups, indicating that pre-pregnancy BMI had a crucial impact on the blood glucose of pregnant women, with blood glucose increasing proportionately with an increase in BMI. By implementing the comprehensive management strategies during the one-day outpatient service, fasting blood glucose in all three groups was decreased, suggesting that the outpatient service had a vital impact on controlling blood glucose and protecting patients with GDM and different pre-pregnancy BMIs from pre-diabetes progressing to diabetes.

According to the 2020 ADA guidelines for the diagnosis and treatment of diabetes during pregnancy, it is appropriate to set the HbA1C target to < 6%. Following a GDM diagnosis, HbA1C in the three groups was in the normal range; however, the low weight group showed the lowest HbA1C level compared with the normal weight and overweight and obesity groups. The data also showed that changes in blood glucose proportionally increased along with BMI. However, this study found that from enrollment to delivery, the average level of HbA1C increased in all three groups, with no significant difference in the degree of increase among the three groups, and HbA1C was within the normal range. In this case, however, HbA1C reflected only the average blood glucose levels of the previous 2–3 months. Additionally, insulin resistance in pregnant women with GDM was mild during early pregnancy but gradually increased at 24–28 weeks of pregnancy. Following a diagnosis, insulin resistance was steadily aggravated. Furthermore, the 2017 China Diabetes Society guidelines note that HbA1c was often underestimated and had limited value in GDM diagnosis because of increased red blood cell conversion in the second and third trimesters, and because of the effects of anemia during pregnancy.

The insulin utilization rate was different among the three groups; the overweight and obesity group showed the highest insulin utilization rates. Among the three groups, the overweight and obesity group presented with the highest blood glucose levels. As a result of the comprehensive management presented in the one-day outpatient service, the blood glucose levels of these participants decreased compared with their initial levels; however, the overweight and obesity group still reported the highest levels of insulin utilization. These findings highlight that an increased BMI corresponds to greater perturbations in blood glucose and subsequently increases the likelihood that insulin will be used as a treatment for controlling blood glucose during pregnancy. Moreover, studies have shown that a pre-pregnancy BMI has a greater impact on insulin resistance than weight gain during pregnancy, with a higher BMI before pregnancy correlating with more severe insulin resistance (20). Other studies have also demonstrated that insulin resistance indices were elevated at the time of a GDM diagnosis in patients who had been obese before conception, and the insulin resistance indices were positively correlated with BMI both before pregnancy and at GDM screening (21). Therefore, it may be more challenging to control blood glucose during pregnancy in patients who had been overweight or obese before becoming pregnant due to more severe insulin resistance and a subsequent increase in the use of insulin.

In this study, the overweight and obesity group had the highest incidence of gestational hypertension/preeclampsia and neonatal jaundice, indicating that being overweight or obese prior to becoming pregnant may increase the incidence of hypertension and neonatal jaundice in patients with GDM. No significant difference was observed in the rates of premature membrane rupture, premature delivery, polyhydramnios/oligohydramnios, neonatal hypoglycemia, fetal distress, macrosomia, fetal growth restriction, or cesarean section. These findings may have resulted from = improved blood glucose and weight control as a result of attending the one-day outpatient service.

In addition, the study results showed that positive (but not excessive) weight gain and normal weight gain were predominant among the three groups, and the differences were not statistically significant. However, the overweight and obesity group had the highest rate of excessive weight gain, and the differences among the three groups were statistically significant. The highest negative weight-gain rate and the no weight-gain rate were observed in the overweight and obesity group, followed by the normal weight group, with the lowest rates found in the low-weight group. These data indicated that excessive weight gain in the overweight and obesity group occurred prior to enrollment, but the weight gain had been well-controlled after comprehensive diabetes outpatient management. Excessive weight gain might occur during pregnancy because of excessive weight gain that occurred prior to enrollment. Excessive weight gain during pregnancy is associated with an increased risk of medication use, hypertensive disorders, cesarean section, gestational age, and macrosomia, compared with normal or no excessive weight gain. Furthermore, low weight gain during pregnancy was shown to have a protective function against macrosomia and did not increase the risk of low birth weight. In pregnant women with GDM, less weight gain than what is recommended is beneficial, but the effective prevention of excessive weight gain is paramount (22).

After recruitment to the one-day outpatient service, some pregnant women evidenced no weight gain, as well as negative weight gain, but only three pregnant women showed negative weight gain during the entire pregnancy period, which was considered to have been related to a high pre-pregnancy BMI and excessive weight gain before a GDM diagnosis. After delivery of the outpatient service, no statistically significant difference was observed in the incidence of hypertension/preeclampsia, premature rupture of membranes, chorioamnionitis, premature delivery, polyhydramnios/oligohydramnios, neonatal jaundice, neonatal hypoglycemia, fetal distress, macrosomia, fetal growth restriction, and cesarean section rate among the participants with a negative, zero, and positive weight gain (*P* > 0.05). The results showed that no weight gain and negative weight gain had no significant adverse effects on maternal and infant outcomes. Existing findings have demonstrated a higher gestational weight gain during the third trimester (28–36 weeks) to be associated with LGA babies, higher insulin doses, and increased postpartum 2-h OGTT results (23). Therefore, the one-day outpatient service had a substantial impact on weight control in pregnant women who had been diagnosed with GDM, as demonstrated by the controlled weight gain in late pregnancy and the reduced occurrence of related complications.

Collectively, the current study results showed that the comprehensive one-day outpatient management of DM may control blood glucose and body weight in GDM patients with different pre-pregnancy BMIs. Overweight or obese patients reflected a higher risk of developing gestational hypertension/preeclampsia and neonatal jaundice. Furthermore, these patients had a higher use rate of insulin for controlling blood glucose. Some pregnant women showed negative weight gain, no weight gain, or insufficient weight gain, but these outcomes did not increase the risk of adverse pregnancy outcomes; this may have been due to inappropriate weight gain before or during early pregnancy. Therefore, women of childbearing age should control their weight to ensure they remain within a reasonable weight range before becoming pregnant to reduce the risk of GDM.

The current study includes several limitations. First, all of the participants were recruited from the authors' hospital, thus limiting the patient representation. Second, all patient information was collected through the electronic medical records system of the authors' hospital, but, some important measurements were not included. Third, since this had been a cohort study, the authors lost some test information from participants in the mid-phase of the study. Nevertheless, despite these limitations, the authors can confidently assert that the one-day outpatient service presents a promising and effective strategy for improving the current management of women with GDM.

## Data availability statement

The original contributions presented in the study are included in the article/supplementary material, further inquiries can be directed to the corresponding authors.

## Ethics statement

The studies involving human participants were reviewed and approved by The Fourth Hospital of Shijiazhuang Ethics Committee. The patients/participants provided their written informed consent to participate in this study.

## Author contributions

Conception and design of the research, acquisition of data, and writing of the manuscript: Y-MC and MM. Analysis and interpretation of the data: MM and WW. Statistical analysis and critical revision of the manuscript for intellectual content: WW and N-NC. All authors have read and approved the final draft.
